# Face-to-Face Sharing with Strangers and Altruistic Punishment of Acquaintances for Strangers: Young Adolescents Exhibit Greater Altruism than Adults

**DOI:** 10.3389/fpsyg.2016.01512

**Published:** 2016-10-03

**Authors:** Jian Hao, Yue Yang, Zhiwen Wang

**Affiliations:** ^1^Beijing Key Laboratory of Learning and Cognition, Department of Psychology, College of Education, Capital Normal UniversityBeijing, China; ^2^School of Nursing, Peking UniversityBeijing, China

**Keywords:** sharing, altruistic punishment, third-party punishment, fairness, adolescent

## Abstract

Young adolescents are generally considered to be self-absorbed. Studies indicate that they lack relevant general cognitive abilities, such as impulse control, that mature in early adulthood. However, their idealism may cause them to be more intolerant of unfair treatment to others and thus result in their engaging in more altruistic behavior. The present study aimed to clarify whether young adolescents are more altruistic than adults and thus indicate whether altruistic competence is domain-specific. One hundred 22 young adolescents and adults participated in a face-to-face, two-round, third-party punishment experiment. In each interaction group, a participant served as an allocator who could share money units with a stranger; another participant who knew the allocator could punish the acquaintance for the stranger. Participants reported their emotions after the first round, and at the end of the experiment, the participants justified their behavior in each round. The results indicated that the young adolescents both shared more and punished more than did the adults. Sharing was associated with a reference to fairness in the justifications, but altruistic punishment was associated with subsequent positive emotion. In sum, greater altruism in young adolescents compared to adults with mature cognitive abilities provides evidence of domain-specificity of altruistic competence. Moreover, sharing and altruistic punishment are related to specific cognitive and emotional mechanisms, respectively.

## Introduction

Prosocial behavior is important for the quality of interactions between the self and others (Eisenberg et al., 2006). Studies have found that children’s prosocialness positively predicts their adolescent social preferences as well as their academic achievement (Caprara et al., 2000). In other words, prosocialness facilitates not only social but also cognitive development.

Altruistic behavior as a significant index of prosocialness has received considerable attention. Traditionally, altruistic individuals engage in specific behavior that benefits others regardless of cost and lack of reward (Bryan and London, 1970; Macaulay and Berkowitz, 1970; Rushton, 1976). For example, sharing and helping behavior are usually considered altruistic behavior. However, altruism is not only manifested in positive behavior, but it is also manifested through punishment. Altruistic punishment refers to punishing others for violating social norms (Fehr and Fischbacher, 2003), and similar to altruistic sharers, there is high cost and little reward for altruistic punishers (Fehr and Gächter, 2002).

There are important theoretical distinctions between sharing and altruistic punishment. According to Kinnunen and Windmann’s (2013), first, sharing involves help giving, whereas altruistic punishment involves fairness and cooperation maintaining; second, sharing involves one party’s giving to another, whereas altruistic punishment can be considered as “a social investment benefiting societal groups at large” (Kinnunen and Windmann, 2013, p. 1). In addition, recipients of sharing are positively treated, but recipients of punishment are negatively treated. Thus, sharing and altruistic punishment are two facets of altruistic behavior.

Previous studies have examined the two facets of altruistic behavior. A number of studies have found sharing behavior in preschool children, and some of them have indicated that sharing increases with age during preschool years (Gummerum et al., 2010; Takagishi et al., 2010, 2014; Paulus et al., 2013; Paulus and Moore, 2014). Moreover, a recent study showed that extrinsic rewards had negative impact on young children’ sharing behavior, providing evidence for their intrinsic altruism (Ulber et al., 2016). From early childhood to middle childhood, children’s sharing continues to develop. Bar-Tal et al. (1980) found that among preschool and elementary children, a higher percentage of the oldest children referred to high-level motives, such as a personal willingness to share, for their sharing behavior. Furthermore, most studies also indicate that the amount of children’s sharing or donating increases with age from early to middle childhood (Skarin and Moely, 1976; Benenson et al., 2007; Ongley et al., 2014). Therefore, previous studies have shown that traditional altruistic behavior, such as sharing, develops quickly in early developmental stages. By contrast, fewer studies find altruistic punishment in young children. Some studies have shown that when confronted with others’ harmful behavior or intentions, young children tattle or avoid helping the actors (Vaish et al., 2010, 2011). It is thus suggested that young children are sensitive to violation of moral norm. Recent studies further conclude that young children are able to carry out altruistic punishment (Kenward and Östh, 2015; McAuliffe et al., 2015). However, young children may punish, within limits. For example, they punish less when they have to do so in person (Kenward and Östh, 2015) or when there is a cost associated with punishment (McAuliffe et al., 2015).

By contrast, altruistic punishment is more typically exhibited among adults. For example, in Fehr and Gächter’s (2002) public goods game, participants could punish other members when they knew the others’ investments. The researchers found that most adults imposed punishment at least once. Additionally, Fehr and Fischbacher (2004) introduced a third party in the dictator game wherein the third parties could punish allocators after they witnessed the allocators’ distributing resources between themselves and the receivers. The results indicated that more than half of the third parties punished the allocators when the distributions were unfair. Sun et al. (2015) further reported that adults with high altruistic tendencies carried out more third-party punishments than did those with low altruistic tendencies in unfair situations. Overall, altruistic punishment is more common in adults (Henrich et al., 2006).

As sharing and altruistic punishment are two facets of altruistic behavior, it is necessary to examine altruism from the two facets of altruistic behavior. Sharing with strangers may better reflect altruistic behavior because people seem more unwilling to share with strangers. Previous study found that between the ages of 3 and 8 years, children’s choices of sharing with a partner increased when the partner was an in-group child but decreased when the partner was an out-group child (Fehr et al., 2008). However, altruistic punishment of strangers may not better reflect altruistic behavior. Individuals are able to punish strangers in part because strangers are unrelated people whom the punisher will likely never meet again. By contrast, altruistic punishment of acquaintances may present genuine pressure and thus be a stronger evidence of altruism. Studies also confirm that out-group members are punished more strongly than are in-group members (Jordan et al., 2014; Schiller et al., 2014). Accordingly, altruistic behavior may be better revealed by examining sharing with strangers and altruistic punishment of acquaintances for strangers. These two types of altruistic behavior benefit strangers in different ways. Finally, in previous studies, participants implemented punishment via computers, and thus, they did not directly interact with each other (e.g., Fehr and Gächter, 2002). Given that most interactions occur face-to-face, real interactions in altruistic situations guarantee greater ecological validity.

Young adolescents’ altruism with respect to the two facets of altruistic behavior is not clear, and it is theoretically important to clarify this issue. First, there is a significant change in adolescents’ perspectives regarding altruism-related themes such as fairness (Crone, 2013). Adolescents have acquired abstract thinking in the formal operational stage (Inhelder and Piaget, 1958). On the one hand, as the abstract thoughts result in self-absorption, a key characteristic of early adolescence (Crone, 2013), young adolescents may become less concerned about justice and fairness, and thus become less altruistic. On the other hand, the abstract thoughts cause idealism, with adolescents imagining an ideal world without injustice and unfairness (Berk, 2005). As a result, they may become less tolerant of unfair treatment of others, and thereby become more altruistic. The two possible changes imply that adolescence is a critical period with respect to the development of altruism. Therefore, it is necessary to clarify whether young adolescents are more altruistic or less altruistic. However, young adolescents’ self-absorption should not be equated with egocentrism, a characteristic of young children. Their self-absorption does not mean that they cannot understand others’ thoughts and feelings. Young adolescents perform as well on theory of mind tasks as young adults (e.g., Hao and Liu, 2016). Therefore, self-absorption may not necessarily result in lack of altruism, but idealism may facilitate altruism.

Second, differences in altruism between young adolescents and adults will help clarify whether altruistic competence is domain-general or domain-specific. There may be different domains regarding people’s cognition about the world and the corresponding performance. According to perspectives of domain-specificity, “the mind is in some sense compartmentalized or ‘modularized’; that is, that human conceptual understanding of one sort (e.g., about space) is likely to be quiet different in character, structure, and development from understanding of another sort (e.g., about language)” (Wellman and Gelman, 1992, p. 338). Perspectives of domain-generality emphasizes that one domain is similar to another in the above aspects. Thus, if altruistic competence is different from general cognitive ability in development, altruistic competence is likely to be domain-specific. If altruistic competence is similar to general cognitive ability in development, it may be domain-general. Executive function is an important general cognitive ability as it is useful for goal-directed problem solving (Hongwanishkul et al., 2005). One of its key components, impulse control (Anderson, 2008), is considered to be related to concern for others (Crone, 2013), because inhibition of selfish impulse enables individuals to consider others’ benefit in decision-making and thus carry out altruistic behavior. According to Crone’s (2013) model, which is based on empirical studies, young adolescents are inferior to young adults in impulse control. Thus, superior altruistic competence in young adolescents indicates that development of altruistic competence is different from that of impulse control, suggesting domain-specificity of altruistic competence. Inferior altruistic competence in young adolescents indicates that altruistic competence develops with impulse control, suggesting domain-generality of altruistic competence.

Previous studies have shown that infants display altruistic behavior to some extent even though their general cognitive ability has not matured. Warneken and Tomasello (2006) found that 18-month-old infants could help adults to achieve their goals. Schmidt and Sommerville (2011) reported that 15-month-old infants were able to share with adults. Thus, infants may have a naturally altruistic tendency (Warneken and Tomasello, 2009). Because altruistic punishment is beneficial to cooperation (Fehr and Gächter, 2002), punitive sentiment as a domain-specific mechanism may evolve (Kenward and Östh, 2015). Schmidt and Sommerville (2011) found that infants as young as 15 months of age looked longer to the unfair allocation than the fair one, which implies their sensitivity to fairness. Taken together, previous studies indicate that altruistic competence may be domain-specific to some extent.

The proximate mechanisms of sharing and altruistic punishment also require further clarification. Some researchers maintain that cognitive processes influence moral behavior (Kohlberg, 1969), whereas others argue that emotional processes play an important role in moral behavior (Hoffman, 2000; Eisenberg, 2010; Eisenberg et al., 2010). Because allocators must distribute resources between themselves and others, it inevitably involves consideration of fairness. Thus, sharing may engage cognitive processes. However, realizing unfairness may not necessarily result in altruistic punishment. Altruistic punishment is likely to be associated with emotional arousal because individuals tend to avoid treating others negatively unless their emotions are aroused and are difficult to control. Fehr and Gächter (2002) also proposed that negative emotions toward free-riders trigger altruistic punishment. If that is the case, punishers should experience increased positive emotions after they have imposed stronger altruistic punishment.

In sum, the present study aimed to clarify two issues. First, do young adolescents exhibit more altruistic behavior than adults? Second, are sharing and altruistic punishment related to specific cognitive and emotional processes, respectively? Fehr and Fischbacher’s (2004) third-party punishment paradigm was used because it involved both sharing and altruistic punishment. The experiment was conducted in two rounds. Participants’ sharing or altruistic punishment in the two rounds would be compared. This analysis aimed to clarify whether altruistic tendencies in young adolescents or adults were stable. In each round, a stranger (experiment confederate) and two participants who knew each other interacted face-to-face. A participant as an allocator decided how to share an amount of money units (MUs) with the stranger. After allocation, another participant as a punisher could punish the allocator, i.e., the acquaintance, for the stranger. At the end of the first round, participants’ emotions were assessed. The second round was then begun. Finally, participants were asked to justify their behavior in each round. Participants’ justifications were coded with respect to their consideration of fairness. According to previous studies, two hypotheses were proposed. First, young adolescents outperform adults in both sharing and altruistic punishment. Second, sharing is associated with consideration of fairness, whereas altruistic punishment is related to emotional arousal.

## Materials and Methods

### Participants

A total of 122 participants took part in the present study. The young adolescent group consisted of 66 participants, 33 males and 33 females, whose ages were between 13.00 and 13.92 years of age (*M* = 13.56, *SD* = 0.28). All adolescents were recruited from a middle school in Beijing. The adult group consisted of 56 participants, 26 males and 30 females, who ranged in age from 18.00 to 32.67 years (*M* = 20.95, *SD* = 2.79). The adults were recruited from a university in Beijing and were either undergraduate or graduate students. The study was approved by the Research Ethics Board of the Department of Psychology of Capital Normal University. Informed written consent was obtained from all participants.

### Materials and Procedure

#### Third-Party Punishment

The third-party punishment experiment was adapted from Fehr and Fischbacher’s (2004) paradigm. In each group, two participants who knew each other were randomly assigned the role of allocator or punisher; the experiment confederate was a stranger to the two participants and thus was assigned the role of receiver. Therefore, the allocator could share with the stranger, and the punisher could punish the allocator for the stranger in each group. Specifically, the allocator was given 10 pieces of model paper money, i.e., 10 MUs. Each MU represented 10 yuan. The allocator could allocate a certain amount of MUs to the stranger according to his/her willingness. The punisher was given five MUs and could punish the allocator according to his/her willingness. If the punisher paid one MU, the allocator would lose three MUs. All participants clearly knew how they were to interact in the group. According to previous studies (Brüne et al., 2012; Sun et al., 2015), the MUs the allocator lost and the MUs the punisher paid were finally transferred to the stranger. Thus, the participants knew that the MUs they lost or paid were used for altruistic rather than other purposes. The number of MUs the allocators shared with the stranger represented their sharing levels. The number of MUs the punishers paid represented their altruistic punishment levels.

#### Emotion Rating

At the end of the first round, participants were presented with a paper that contained 20 words, each of which represented an emotion. The emotion words consisted of three categories, including positive words (e.g., happy), negative words (e.g., angry) and neutral words (e.g., calm), that were arranged randomly in a 4 × 5 matrix. Participants were then individually asked to choose the word from the matrix that best described their emotion at that moment. If they chose positive or negative words, they were then asked to rate the intensity of their emotions on a scale from 1 (a little) to 5 (very much). The scores for negative words were -5 to -1, and the scores for positive words were 1 to 5. The scores for neutral words were 0. Accordingly, participants’ emotion scores ranged from -5 to 5.

#### Justifications

At the end of the second round, participants were individually asked why they gave certain amounts of MUs in each round. Answers with reference to fairness in each round scored a 1. For example, allocators’ justifications scored a 1 if they articulated justifications such as “to keep it fair.” Punishers’ justifications scored a 1 if they articulated justifications such as “to punish the unfair behavior.” The other unrelated answers scored a 0. Accordingly, participants’ fairness scores ranged from 0 to 2 for the two rounds. Another rater who did not know the aim of the experiment rated the justifications of 20% of the participants. Inter-rater reliability was good with kappa = 1.00.

## Results

### Altruistic Behavior of Young Adolescents and Adults in the Two Rounds

**FIGURE 1:**
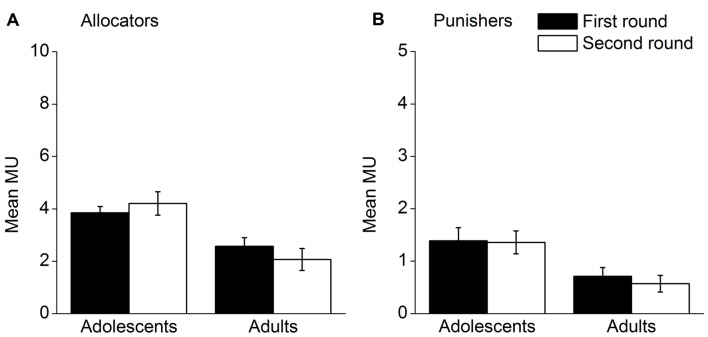
**Altruistic behavior in the two rounds for each age group; (A) mean MUs allocators shared, (B) mean MUs punishers used.** Error bars represent standard error.

Altruistic behavior of young adolescents and adults in the two rounds were analyzed. Mean MUs the allocators shared and mean MUs the punishers used in the two rounds for each age group are presented in **Figure 1**. With respect to sharing (**Figure 1A**), a 2 (round) × 2 (age group) repeated measures ANOVA did not find a significant interaction effect of round and age group, *F*(1,59) = 2.54, *p* = 0.116, η^2^ = 0.041. There was also no significant main effect of round, *F*(1,59) = 0.06, *p* = 0.802, η^2^ = 0.001. The allocators performed similarly in the two rounds. A main effect of age group was found to be significant, *F*(1,59) = 14.29, *p* < 0.001, η^2^ = 0.195. The adolescent allocators shared more MUs than the adult allocators. With regard to altruistic punishment (**Figure 1B**), a 2 (round) × 2 (age group) repeated measures ANOVA was conducted. The results were similar to those of sharing. No significant interaction effect was found, *F*(1,59) = 0.13, *p* = 0.722, η^2^ = 0.002. The main effect of round was not significant, *F*(1,59) = 0.30, *p* = 0.584, η^2^ = 0.005. The punishers performed similarly in both rounds. However, there was a main effect of age group, *F*(1,59) = 8.35, *p* = 0.005, η^2^ = 0.124. The adolescent punishers used more MUs to punish the allocators than did the adult punishers. Accordingly, young adolescents displayed more altruistic behavior than adults across the two rounds.

### Altruistic Behavior of Males and Females in the Two Rounds

**FIGURE 2:**
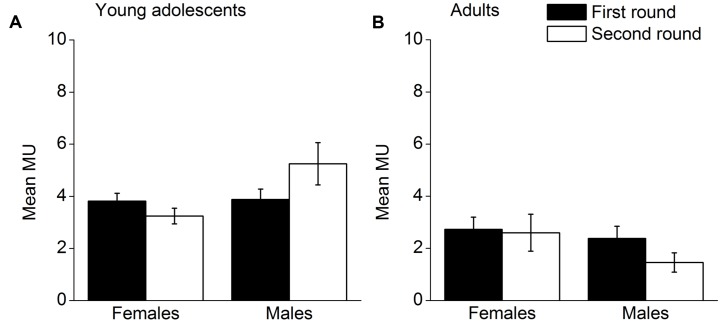
**Sharing in the two rounds for males and females; (A) mean MUs adolescent allocators shared, (B) mean MUs adult allocators shared.** Error bars represent standard error.

Altruistic behavior in the two rounds for males and females was analyzed. Mean MUs allocators shared by round, gender and age group are presented in **Figure 2**. A 2 (round) × 2 (gender) × 2 (age group) repeated measures ANOVA obtained a significant interaction effect of the three variables, *F*(1,57) = 7.20, *p* = 0.010, η^2^ = 0.112. Therefore, a separate 2 (round) × 2 (gender) repeated measures ANOVA was conducted for each age group. With respect to young adolescents (**Figure 2A**), the interaction effect of round and gender was significant, *F*(1,31) = 7.14, *p* = 0.012, η^2^ = 0.187. A *t*-test indicated that the number of MUs females shared significantly decreased from round 1 to round 2, *t*(16) = 2.28, *p* = 0.037, but the number of MUs males shared had a tendency to increase from round 1 to round 2, *t*(15) = -1.95, *p* = 0.071. With respect to the adults (**Figure 2B**), the interaction effect of round and age group was not significant, *F*(1,26) = 1.27, *p* = 0.269, η^2^ = 0.047. The number of MUs females shared did not change from round 1 to round 2, *t*(14) = 0.24, *p* = 0.815. The number of MUs males shared decreased from round 1 to round 2, *t*(12) = 2.41, *p* = 0.033. In other words, adolescent males were inclined to share more after the first round, whereas adolescent females and adult males shared less after the first round.

**FIGURE 3:**
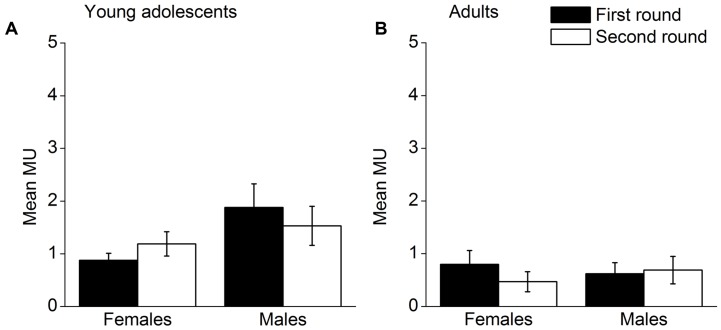
**Altruistic punishment in the two rounds for males and females; (A) mean MUs adolescent punishers used, (B) mean MUs adult punishers used.** Error bars represent standard error.

Mean MUs punishers used by round, gender and age group are presented in **Figure 3**. A 2 (round) × 2 (gender) × 2 (age group) repeated measures ANOVA was conducted. There was no significant interaction effect of the three variables, *F*(1,57) = 2.98, *p* = 0.090, η^2^ = 0.050. The only significant effect is the main effect of age group (**Figure 3A** for young adolescents’ results and **Figure 3B** for adults’ results), *F*(1,57) = 8.35, *p* = 0.005, η^2^ = 0.128. These results indicate that altruistic punishment afforded by males and females was stable across the two rounds for both age groups.

### Relationships between Altruistic Behavior and Emotional and Cognitive Processes

**FIGURE 4:**
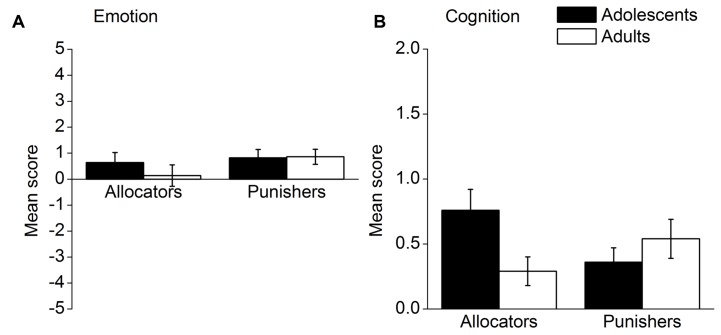
**Mean emotion and cognitive scores by role and age group; (A) mean emotion scores, (B) mean fairness scores.** Error bars represent standard error.

Emotional arousal and consideration of fairness in young adolescents and adults were first analyzed. Mean emotion and cognitive scores by role and age group are presented in **Figure 4**. A 2 (role) × 2 (age group) ANOVA with emotion scores as dependent variable (**Figure 4A**) found no significant interaction effect, *F*(1,118) = 0.56, *p* = 0.455, η^2^ = 0.005. Furthermore, there was neither a main effect of role [*F*(1,118) = 1.59, *p* = 0.210, η^2^ = 0.013] nor a main effect of age group [*F*(1,118) = 0.41, *p* = 0.524, η^2^ = 0.003]. With respect to fairness scores (**Figure 4B**), a 2 (role) × 2 (age group) ANOVA revealed a significant interaction effect, *F*(1,118) = 5.58, *p* = 0.020, η^2^ = 0.045. A *t*-test illustrated that adolescent allocators more frequently mentioned fairness in their justifications than did adult allocators, *t*(59) = 2.44, *p* = 0.018. However, there were no significant differences in fairness consideration between adolescent and adult punishers, *t*(59) = -0.93, *p* = 0.356. Therefore, from the group level, adolescent allocators considered fairness more often than did the adult allocators.

Further analyses were conducted to explore whether individual differences in cognitive and emotional processes were associated with individuals’ altruistic behavior. Separate correlation analyses were performed for young adolescents and adults, respectively. With respect to young adolescents, allocators’ shared MUs in round 1 were significantly correlated with their fairness scores, *r* = 0.57, *p* = 0.001, and MUs punishers used in round 1 were significantly correlated with their emotion scores, *r* = 0.40, *p* = 0.023. Furthermore, there is a marginally significant correlation between MUs punishers used in round 1 and MUs allocators shared in round 2, *r* = 0.32, *p* = 0.065. The results for adults were similar, to some degree, to those for young adolescents. Allocators’ shared MUs in round 1 and 2 were, respectively, significantly associated with their fairness scores, *r* = 0.58, *p* = 0.001; *r* = 0.40, *p* = 0.036. MUs punishers used in round 1 were significantly correlated with their emotion scores, *r* = 0.59, *p* = 0.001. However, there was a tendency that the more MUs the punishers used in round 1, the fewer MUs the allocators shared in round 2, *r* = -0.32, *p* = 0.096. Moreover, there was a significant negative correlation between MUs punishers used in round 1 and allocators’ emotion scores, *r* = -0.58, *p* = 0.001. Taken together, the results indicated that for both age groups, sharing was associated with fairness consideration, whereas altruistic punishment was associated with emotional arousal. In addition, young adolescents seemed to respond positively toward others’ punishment, whereas adults’ reactions were more negative.

## Discussion

The present study examined sharing with strangers and altruistic punishment of acquaintances for the strangers in face-to-face interactions in young adolescents and adults. The study yielded some new findings. First, the young adolescents both shared more and punished more than did the adults. Furthermore, the adolescent male allocators were inclined to share more after the first round, whereas the adolescent females and adult males shared less after the first round. Second, sharing was associated with consideration of fairness, but altruistic punishment was related to emotional arousal.

Sharing and altruistic punishment are two facets of altruistic behavior, but there are some differences between them. Because sharing involves positive treatment of others, individuals are likely to have positive feelings or anticipation of positive feelings. Previous studies are consistent with this perspective. Aknin et al. (2015) found that for both preschool children and adults, giving treats to others brought them more happiness than receiving treats themselves. Paulus and Moore (2016) recently confirmed that even preschoolers could expect positive emotions produced by sharing. By contrast, although altruistic punishment is also aimed at benefiting others, the goal must be achieved through negative treatment of the allocator. In the present study, the allocators were also the acquaintances of the punishers. Thus, altruistic punishment may require greater courage, especially moral courage. In fact, altruistic punishment is considered as a moral courage situation (Kinnunen and Windmann, 2013). In addition, sharing involves benefiting others directly. In the situation of altruistic punishment, especially third-party punishment, the punishers as the observers do not directly experience allocation (Kenward and Östh, 2015). Thus, the third-party punishers must intervene in the allocation in order to carry out altruistic punishment.

Although the present study examines behavioral altruism such as sharing, the motivation for sharing may be open to question. According to the theory of direct reciprocity (Trivers, 1971), one person shares with or helps the other due to the expectation of the similar treatment by the other. Direct reciprocity may exist when the same two persons have repeated interactions and both of them are able to help (Nowak, 2006). In the present study, only the allocator could decide how to share and the receiver had no opportunity to do so. Moreover, because the receiver is a stranger to the allocator, the possibility of their future encounter is very small. Thus, direct reciprocity may not explain participants’ sharing in the present study. The theory of indirect reciprocity emphasizes that one can benefit from his/her reputation in the future, which is established through his/her current altruistic behavior (Nowak and Sigmund, 1998; Leimar and Hammerstein, 2001). Thus, participants may share in order to gain reputation. In addition, escape from punishment may be another possible reason for sharing. However, further analysis showed that only some participants made at least fair allocation which helps acquire reputation and escape punishment. Therefore, reputation gain or escape from punishment may be not the primary reasons for participants’ sharing. Their sharing may reflect their altruistic willingness to some extent. However, these strategic reasons for altruism may not be fully ruled out. Some studies have indicated that young children are sensitive to their reputation (Leimgruber et al., 2012; Engelmann et al., 2013). Therefore, sharing behavior needs to be better assessed to rule out these possibilities in future studies.

More importantly, the present study showed that the young adolescents exhibited more sharing and altruistic punishment than did the adults. According to Crone’s (2013) model, individuals in early adolescence are more self-oriented, probably because they are unable to control selfish impulse. Nonetheless, the present study found that these adolescents were not less altruistic than adults. In Güroǧlu et al.’s (2009) study, when confronted with a fair versus an unfair allocation, 12-year-old adolescents refused the unfair offers as did the adults. These researchers further found that 12-year olds displayed similar levels of prosocial behavior toward friends, antipathies, neutral peers and anonymous peers, while older adolescents’ prosocial behavior was more influenced by their relationships with the partners (Güroğlu et al., 2014). This implies that young adolescents may have a greater of fairness than older adolescents. In addition, Zanolie et al. (2015) reported that the rejection of an unfair offer depended on whether the alternative was a fair or a hyper-fair offer, and the decision pattern was similar for both mid-adolescents and adults. Accordingly, empirical studies suggest that young adolescents seem not only comparable to adults but also superior to them with respect to altruistic behavior.

Greater altruism in young adolescents not only reflects altruistic characteristics in early adolescence, but also reveals domain-specificity of altruistic competence to some extent. General cognitive abilities tend to increase with age from childhood to early adulthood. For example, an important component of executive function, inhibitory control, displays such universal developmental trend (Williams et al., 1999; Bedard et al., 2002). However, the present study found that the young adolescents were more altruistic than the adults. From early adolescence to early adulthood, the different developmental trend in altruistic competence suggests that altruistic competence may be domain-specific at least during this developmental period. In addition, previous studies have indicated that adolescents experience some specific changes. First, adolescents become idealistic. For example, Thornbug et al. (1984) asked adolescents to rank 18 values according to importance. World peace was ranked fourth by 9th-grade adolescents, a result explained by their modality for idealism. Second, after early adolescence, individuals’ self-esteem begins to increase (Twenge and Campbell, 2001). Third, morality and identity possibly become integrated during adolescence (Moshman, 2011), thus resulting in the development of moral identity. Moral identity refers to “the extent to which people identify with, and are invested in, being a moral person and doing what is moral” (Hardy et al., 2014, p. 45). These specific changes enable young adolescents to expect a world of fairness and peace and to strive to be morally good. In other words, although young adolescents do not have mature general cognitive ability, these specific developmental characteristics can still facilitate their altruistic behavior. Therefore, altruistic competence may be domain-specific to some extent.

Furthermore, the present study also found gender differences regarding altruistic behavior. Adolescent male allocators had a tendency to share more after round 1, a pattern that was reversed for adolescent females and adult males. According to Gilligan’s (1982) perspective, males place greater value on justice, whereas females place greater value on care. Meanwhile, adults may be more economical. Novakova and Flegr (2013) reported that there are negative relationships between the amount at stake and adults’ proposed shares in the dictator and ultimatum games. Thus, adults are more likely to consider the cost of fairness. Taken together, increase in fairness-based altruism from round 1 to round 2 was exhibited by adolescent males.

The proximate mechanisms of altruistic competence were further explored in the present study. The results indicated that sharing was associated with consideration of fairness. The role of reasoning and reflection in moral development has been confirmed. For example, previous studies have found positive relationships between moral judgments and prosocial behavior (Rubin and Schneider, 1973; Eisenberg et al., 1987, 1991; Ma, 2003). Because sharing involves decisions regarding the balancing of the interests of selves and others, it depends on deliberation. However, altruistic punishment may be based on a different mechanism. Fehr and Gächter (2002) found that norm violators triggered others’ negative emotions. In Buckholtz and Marois’s (2012, p. 659) neurocognitive hypothesis for third-party punishment, amygdala is considered to “generate an affective arousal signal based on the magnitude of the accused’s harm, which may be used as a heuristic to guide punishment severity.” Consistent with these studies, the present study further found that the more MUs punishers paid, the more positive emotions they had. Punishment may provide an outlet for negative emotions toward norm violators and thus cause a positive state. In sum, sharing and altruistic punishment are associated with specific cognitive and emotional mechanisms, respectively.

There was a limitation in the present study. The experiment confederate as the receiver was the same adult stranger for the adolescent and adult groups so that the characteristics of the stranger were controlled in the two groups. Therefore, the stranger was not age-matched for the adolescent group and the present results were based on adolescents’ interaction with adult strangers. Adolescent strangers need to be incorporated in future studies in order to further clarify whether adolescents also exhibit greater altruism toward strangers of their own age.

## Conclusion

The present study indicates that young adolescents both share more and punish more than do adults. Greater altruism exhibited by young adolescents compared to that exhibited by adults with mature cognitive abilities provides evidence of domain-specificity of altruistic competence. Moreover, fairness consideration is the specific cognitive mechanism of sharing, whereas emotional arousal is the specific emotional mechanism of altruistic punishment.

## Author Contributions

JH proposed the concept and designed the work; YY performed the acquisition of data for the work; JH and ZW analyzed and interpreted the data. JH drafted the work; JH, YY, and ZW revised the work for important intellectual content. All authors finally approved the version to be published. All the authors agreed to be accountable for all aspects of the work in terms of ensuring that questions related to the accuracy or integrity of any part of the work are appropriately investigated and resolved.

## Conflict of Interest Statement

The authors declare that the research was conducted in the absence of any commercial or financial relationships that could be construed as a potential conflict of interest.
